# Design of Low-Density Parity-Check Code Pair for Joint Source-Channel Coding Systems Based on Graph Theory

**DOI:** 10.3390/e25081189

**Published:** 2023-08-10

**Authors:** Yijie Lv, Jiguang He, Weikai Xu, Lin Wang

**Affiliations:** 1 Department of Information and Communication Engineering, Xiamen University, Xiamen 361005, China; lvsejianke@126.com (Y.L.); wanglin@xmu.edu.cn (L.W.); 2 Technology Innovation Institute, Abu Dhabi P.O. Box 9639, United Arab Emirates; jiguang.he@tii.ae; 3 Centre for Wireless Communications, University of Oulu, 90014 Oulu, Finland

**Keywords:** joint source-channel coding, low-density parity-check code, graph theory

## Abstract

In this article, a graph-theoretic method (taking advantage of constraints among sets associated with the corresponding parity-check matrices) is applied for the construction of a double low-density parity-check (D-LDPC) code (also known as LDPC code pair) in a joint source-channel coding (JSCC) system. Specifically, we pre-set the girth of the parity-check matrix for the LDPC code pair when jointly designing the two LDPC codes, which are constructed by following the set constraints. The constructed parity-check matrices for channel codes comprise an identity submatrix and an additional submatrix, whose column weights can be pre-set to be any positive integer numbers. Simulation results illustrate that the constructed D-LDPC codes exhibit significant performance improvement and enhanced flexible frame length (i.e., adaptability under various channel conditions) compared with the benchmark code pair.

## 1. Introduction

A joint source-channel coding (JSCC) system is capable of overcoming the shortcomings of a Shannon separation coding system and enhancing the performance of the digital communication system via joint decoding [1]. In general, a JSCC system can be realized with different combinations of source and channel codes. For instance, examples of these combinations are a variable-length code (VLC) cascaded with a convolutional code (CC) [2,3]; a VLC cascaded with a Turbo code [4,5,6]; a VLC cascaded with a low-density parity-check (LDPC) code [7,8]; and an LDPC code (falling into the category of fixed-length codes) cascaded with another LDPC code [9,10], termed as double LDPC (D-LDPC) codes.

Among these realizations, the D-LDPC code-oriented JSCC system has been intensively investigated since its first appearance in [9,10], where a joint extrinsic information transfer (JEXIT) chart was employed to enhance the waterfall performance of the code pair (one LDPC code for source code and another LDPC code for channel code). To reduce the hardware implementation complexity of the JSCC system and to improve its performance, double protograph LDPC (DP-LDPC) codes for JSCC were extensively studied in [11,12,13,14,15,16]. The source protograph LDPC codes were optimized to improve the error floor of the JSCC system by maximizing the source decoding threshold [12]. Increasing the mutual information (MI) among the source code and the channel code by redesigning the channel protograph LDPC codes leads to performance improvements in the waterfall region [13]. To reduce the transmission latency and decoding complexity, a concatenation of spatially coupled LDPC codes with sliding window decoding for JSCC was proposed in [17]. However, the aforementioned JSCC systems heavily rely on analyses of protograph LDPC codes and JEXIT. Moreover, the candidate frame length of the JSCC based on DP-LDPC code must be restricted to an integer fold of the column number of the base matrix.

Combinatorial mathematics is an important tool for constructing LDPC codes, which includes the graph-theoretic method, combinatoric designs, finite geometries, and difference sets [18,19,20,21]. As an essential construction method for LDPC codes, the channel LDPC codes constructed with the graph-theoretic method achieve promising performance [22].

In this paper, a construction method of the code pair is developed based on graph theory, which transforms parity-check matrices of the source code and the channel code into inter-set constraint problem and selects the sets which satisfy the constraint. In order to match the parity-check matrix of the source code and the generation matrix of the channel code in the encoding process, the channel code is first constructed during the construction of the code pair via the graph-theoretic method. In contrast to the conventional method, the proposed one can be obtained without constructing the base matrix and performing ‘copy and permute’ operations. Since the proposed channel code takes the systematic form, the generator matrix is easily obtained by transposing the parity-check matrix. In addition, the proposed code pair is constructed with set constraints from the graph-theoretic method, which brings a more adaptive/flexible frame length than the conventional protograph code pair.

## 2. Preliminaries of JSCC

### 2.1. Representations of JSCC

The D-LDPC system consists of two LDPC codes: one for source code and the other for channel code. As a linear block code, a parity-check matrix can be used to express the LDPC code. A systematic LDPC code can be completely characterized by a generator matrix $G\in B^{k\times n}$ with B={0,1}, which corresponds to a parity-check matrix $H\in B^{(n-k)\times n}$ of the form in (1), where $P^{T}$ is the transpose of the matrix P and $I_{n-k}$ is the identity matrix with dimension (n−k)×(n−k).

(1) $$H=\left[I_{n-k}|P^{T}\right]={\left[\begin{array}{ccccccccc} 1 & 0 & 0 & \cdots & 0 & p_{11} & p_{21} & \cdots & p_{k1}\\ 0 & 1 & 0 & \cdots & 0 & p_{12} & p_{22} & \cdots & p_{k2}\\ 0 & 0 & 1 & \cdots & 0 & p_{13} & p_{23} & \cdots & p_{k3}\\ \; & \; & & \vdots & \; & & \; & \vdots & \\ 0 & 0 & 0 & \cdots & 1 & p_{1(n-k)} & p_{2(n-k)} & \cdots & p_{k(n-k)} \end{array}\right]}_{(n-k)\times n}$$

The parity-check matrix of the JSCC system is represented as

(2) $$H_{J}=\left[\begin{array}{cc} H_{S} & H_{L}\\ 0 & H_{C} \end{array}\right],$$

which includes three non-zero submatrices, i.e., $H_{S}$ with dimension $m_{s}\times n_{s}$, $H_{C}$ with dimension $m_{c}\times n_{c}$, and $H_{L}$ with dimension $m_{s}\times n_{c}$. Therefore, the overall dimension of $H_{J}$ is $\left(m_{s}+m_{c}\right)\times \left(n_{s}+n_{c}\right)$. A simple example of a parity-check matrix $H_{J}$ is provided in (3).

(3) $$H_{J}=\left[\begin{array}{cccccccccccc} 1 & 1 & 1 & 1 & 1 & 0 & 1 & 0 & 0 & 0 & 0 & 0\\ 0 & 1 & 0 & 0 & 1 & 0 & 0 & 1 & 0 & 0 & 0 & 0\\ 1 & 0 & 1 & 1 & 0 & 1 & 0 & 0 & 1 & 0 & 0 & 0\\ 0 & 0 & 0 & 0 & 0 & 0 & 1 & 1 & 0 & 1 & 1 & 0\\ 0 & 0 & 0 & 0 & 0 & 0 & 1 & 0 & 1 & 0 & 0 & 1\\ 0 & 0 & 0 & 0 & 0 & 0 & 0 & 0 & 0 & 1 & 1 & 1 \end{array}\right].$$

**Figure 1:**
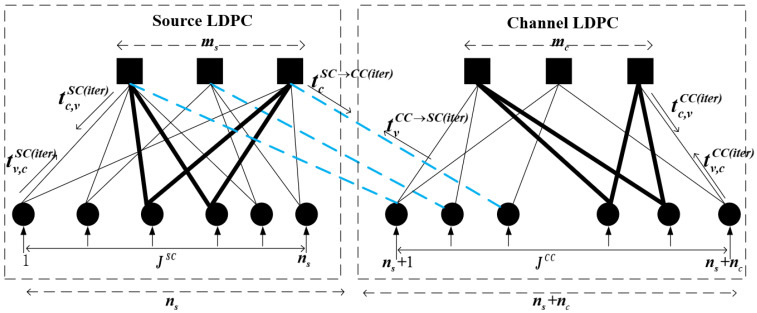
Tanner graphical representation of the JSCC system realized according to exemplary $H_{J}$ in (3).

The Tanner graph associated with the parity-check matrix $H_{J}$ is depicted in Figure 1. Six types of MI in Figure 1 are defined as follows:

* $t_{v,c}^{SC(iter)}$: MI from the variable node (VN) of the source code to the check node (CN) of the source code.
* $t_{c,v}^{SC(iter)}$: MI from the CN of the source code to the VN of the source code.
* $t_{c}^{SC\to CC(iter)}$: MI from the CN of the source code to the VN of the channel code.
* $t_{v}^{CC\to SC(iter)}$: MI from the VN of the channel code to the CN of the source code.
* $t_{v,c}^{CC(iter)}$: MI from the VN of the channel code to the CN of the channel code.
* $t_{c,v}^{CC(iter)}$: MI from the CN of the channel code to the VN of the channel code.

### 2.2. Encoding and Decoding of JSCC Systems

#### 2.2.1. Encoder

The source sequence follows the binomial Bernoulli (*p*) distribution, whose entropy is expressed as

(4) $$H=-p\log_{2}p-\left(1-p\right)\log_{2}\left(1-p\right),$$

with *p* (p≠1/2) being the probability of ‘1’.

The encoding process for the LDPC code pair is represented by

(5) $$c=G_{C}^{T}o=G_{C}^{T}H_{S}s,$$

where $s\in B^{n_{s}}$ denotes the source sequence, while $o\in B^{m_{s}}$ stands for the compressed source sequence. $G_{C}\in B^{\left(n_{c}-m_{c}\right)\times n_{c}}$ is the generator matrix of the channel code. By following the dimension of $H_{S}$, the following constraint

(6) $$n_{c}-m_{c}=m_{s}$$

needs to be satisfied.

The definition of the rate for the low-density parity-check code pair is given by

(7) $$R_{J}=\frac{n_{s}\times \left(n_{c}-m_{c}\right)}{m_{s}\times \left(n_{c}-n_{punc}\right)},$$

where $n_{punc}$ is the number of punctured variable nodes in the channel code.

#### 2.2.2. Decoder

The joint decoding algorithm is applied at the receiver to recover the original source sequence s. The initial source information $J^{SC}$ is denoted by ln((1−p)/p), depending on the source statistics. The initial channel information is denoted by $J^{CC}=2y_{i}/\sigma^{2}$, where $y_{i}=\left(1-2c_{i}\right)+n_{i}$ is the additive white Gaussian noise (AWGN).

## 3. Construction of LDPC Code Pairs

### 3.1. Definitions and Theorems

In Figure 1, the cycles of source and channel codes of the JSCC system in the Tanner graph are underlined with bold lines. The minimum cycle length in a given bipartite graph is the so-called girth of the graph. These cycles directly affect the performance of waterfall and error-floor regions for the LDPC code pair. Particularly, short-length cycles are more detrimental, since the information sent out by the message-passing decoder comes back after a small number of hops to the same node that sent it. This results in biases in the decoding algorithm. All in all, the short-length cycles are particularly deleterious to decoding performance, and thus in the design of the code pair, one should intentionally avoid them. Due to the correspondence between the non-zero elements in the parity-check matrix and the edges in Figure 1, it is possible to avoid short cycles during the construction of the LDPC code pair through structural design. Specifically, each column of the parity-check matrix for both the source and channel codes can be represented by a set, with the row indices of the non-zero elements forming this set. Consequently, short cycles are effectively avoided by designing the relationships between these sets.

A graph consists of vertices (or nodes) and the edges (lines or branches) that connect the vertices. In the sequel, we provide multiple definitions and theorems related to graphs.

**Definition 1.**  *A connected graph, G=(V,E), includes vertex-set $V=\left\{v_{1},v_{2},\ldots \right\}$ with elements called vertices and edge-set $E=\left\{\left(v_{i},v_{j}\right)\right\}$ with pairs of vertices called edges. The end vertices of the edge are the vertices $v_{i}$ and $v_{j}$ that are connected to an edge $\left(v_{i},v_{j}\right)$.*

**Definition 2.**  *Let $T_{1},T_{2},\cdots ,T_{n}$ be n candidate sets, $T_{i}$ contains W elements from {1,⋯,m}, where i∈{1,⋯,n}. Then, for any positive integer t, $\cup^{t}$ denotes the union of t sets selected from the set of all pairwise intersections of the candidate sets, i.e., $T_{i}\cap T_{j}$ for 1≤i,j≤n.*

**Theorem 1.**  *Let E(n) denote a set with n elements and $T_{i},T_{j}$ be subsets of E(n). If $\forall t\in \left\{2,3,\cdots ,s\right\},\max\left(|\cup_{\forall T_{i},T_{j}\subseteq E\left(n\right),i\neq j}^{t}\left(T_{i}\cap T_{j}\right)|\right)<t$ (the union is not calculated when t takes the value 2, i.e., calculating the intersection of $T_{i}$ and $T_{j}$ only), then the cycle length of the Tanner graph is larger than 2s, i.e., the girth of the Tanner is larger than or equal to 2(s+1).*

**Proof.**  When s=2, $\max\left(|(T_{i}\cap T_{j})|\right)<2$, it means that the number of common elements between any two subsets is less than 2. In this case, the cycle length of the Tanner graph exceeds 4. When s>2, an increase in its value means that new connection(s) between the check node and the variable node is added to the Tanner graph. For each additional edge in the Tanner graph, the cycle length and the girth will increase by 2. This is due to the fact that the total of the degrees of the vertices for the graph is twice the number of edges, which implies that the number of common elements between the subsets will increase by 1. That is, if there exists a length-2N cycle, then $t=N,\max\left(|\cup_{\forall T_{i},T_{j}\subseteq E\left(n\right),i\neq j}^{t}\left(T_{i}\cap T_{j}\right)|\right)=N$. For example, if there exists a length-6 cycle, $\exists T_{1},T_{2},T_{3}$ satisfying $|\left(T_{1}\cap T_{2}\right)\cup \left(T_{2}\cap T_{3}\right)\cup \left(T_{1}\cap T_{3}\right)|=3$, which contradicts with Theorem 1 (since according to it, $|\left(T_{1}\cap T_{2}\right)\cup \left(T_{2}\cap T_{3}\right)\cup \left(T_{1}\cap T_{3}\right)|<3$).    □

**Theorem 2.**  *According to Theorem 1, the LDPC code pair is constructed for optimizing the performance of the JSCC system. Each column of the parity-check matrix can be represented by a subset (a subset refers to a group of row indices representing the positions of non-zero elements in a column of the parity-check matrix for the source or channel codes, excluding the identity matrix). The maximum number of columns in the LDPC source code is determined via*

(8) $$P_{s}\leq \left\lfloor \frac{m_{c}\left(m_{c}-1\right)\left({m_{c}}^{2}-m_{c}-{W_{c}}^{2}+W_{c}\right)}{W_{s}{W_{c}}^{2}\left(W_{s}-1\right){\left(W_{c}-1\right)}^{2}}\right\rfloor ,$$

*where the column weights for the source and channel LDPC codes are denoted as $W_{s}$ and $W_{c}$.*

**Proof.**  The parity-check matrix of the systematic channel LDPC code consists of an identity matrix I and a matrix $P_{c}$. The parity-check matrix of the source code is formed with matrix $P_{s}$. The construction of matrix $P_{s}$ and matrix $P_{c}$ are based on Theorem 1. If there does not exist any length-4 cycles, $\forall T_{i},T_{j}\subseteq E\left(n\right),\max(|\left(T_{i}\cap T_{j}\right)\left|\right)<2$, the number of columns of the matrix P is less than or equal to $\frac{m(m-1)}{W(W-1)}$ (the upper bound of row weight of the additional submatrix is $\frac{m-1}{W-1}$), the number of columns of the systematic channel LDPC code is less than or equal to $\frac{m\cdot (W^{2}-W+m-1)}{W\cdot (W-1)}$. (Here, we remove the subscripts for *m* and *W* to make it more general, since this statement can be applied to both source code and channel code.) The condition that a larger-length (>4) cycle does not exist is pre-conditioned on the fact that there does not exist any length-4 circles. Therefore, when a larger-length cycle does not exist, the number of columns of the constructed parity-check matrix has to be reduced to satisfy the stricter set constraint in Theorem 1.    □

### 3.2. Algorithm Description

According to Theorem 1, the corresponding pseudo-code for the proposed construction algorithm of the LDPC code pair is given in Algorithm 1, described as follows:

**Step 1:** Set up the column weight $W_{s}$ of the parity-check matrix for the source code and the column weight $W_{c}$ of the parity-check matrix for the channel code, and calculate their dimensions according to Theorem 2 and (6) (i.e., first calculate the number of columns $n_{s}$ needed in the parity-check matrix of the source code $H_{S}$ based on the corresponding source parameters (e.g., message length and desired code rate). Then, use the desired column weights $W_{s}$ and $W_{c}$, along with $n_{s}$, to calculate the number of rows $m_{c}$ required for the parity-check matrix of the channel code $H_{C}$.

**Step 2:** List all the subsets (of set $\left\{1,2,\cdots ,m_{c}\right\}$) containing $W_{c}$ different elements. The number of such subsets is mcWc.

**Step 3:** Select the desired subsets (from the previous step) satisfying Theorem 1 for constructing the column vector of the parity-check matrix for the channel code $H_{C}$ (the elements in each subset indicate the row indices of ‘1’ in the associated parity-check matrix), which specifies the constraints that the parity-check matrix must satisfy to ensure good error-correction performance.

**Step 4:** Calculate $m_{s}$ according to the relationship in (6) and the constructed channel code from the previous step, and list all the subsets (with $W_{s}$ elements) of set $\left\{1,2,\cdots m_{s}\right\}$ intended for the construction of the parity-check matrix for the source code $H_{S}$.

**Step 5:** According to Theorem 1, select the desired subsets for constructing the column vector of the parity-check matrix for source code $H_{S}$. This matrix must satisfy certain constraints to ensure effective error-correction performance.
**Algorithm 1:** Construction of LDPC code pair**Require:**
  $m_{c}$: row number of source LDPC code
  $W_{s}$: column weight of source LDPC code
  $W_{c}$: column weight of channel LDPC code
  *s*: girth of the constructed source code and channel code.
**Ensure:**
  **Initialize**: parity-check matrix for channel code $H_{C}=\left[I_{m_{c}}\right]$, $n_{c}=m_{c}$. 
1:**Begin**2:$T=\{T_{1},T_{2},\cdots ,T_{i}\}$; ▹ All elements in T are a subset of $\left\{1,\cdots ,m_{c}\right\}$ containing $W_{c}$ elements, i.e.,  $T_{i}=\left\{e_{i,1},\cdots ,e_{i,W_{c}}\right\}$.3:$T_{1}=\left\{e_{1,1},\cdots ,e_{1,W_{c}}\right\}$;4:$H_{C}=\left[H_{C}|h_{T_{1}}\right]$; ▹ The elements in $T_{1}$ indicate the row indices of ‘1’ in the associated column vector $h_{T_{1}}$.5:$n_{c}=n_{c}+1$;6:**for**i=2:mcWc**do**7:   $t_{s}=s$; ▹ If the number of columns in the parity-check matrix is less than $m_{c}+s$, the inter-set constraint conditions will change.8:   **if** $n_{c}-m_{c}+1<s$ **then**9:     ▹ The variable $n_{c}$ represents the number of columns in the parity check matrix $H_{C}$.10:     $t_{s}=n_{c}-m_{c}+1;$11:   **end if**12:   l=0;13:   **for** $t=2:t_{s}$ **do**14:     **if** t=2 **then**15:        $I=\max\left(|\left(T_{j}\cap T_{k}\right)|,\forall T_{j},T_{k}\subseteq E\left(n\right),j\neq k\right),j,k\in \left[1,n_{c}-m_{c}+1\right]$; ▹ Calculate the maximum number of common elements between any two subsets when s=2.16:     **else**17:        $I=\max\left(|\cup_{\forall T_{j},T_{k}\subseteq E\left(n\right),j\neq k}^{t}\left(T_{j}\cap T_{k}\right)|\right),j,k\in \left[1,n_{c}-m_{c}+1\right]$; ▹ Calculate the maximum number of common elements between subsets when s>2.18:     **end if**19:     **if** I<t **then**20:        l=l+1;21:     **else**22:        break;23:     **end if**24:   **end for**25:   **if** $l=t_{s}-1$ **then**26:     ▹ Determine whether the subset $T_{i}$ satisfies **Theorem 1**.27:     $H_{C}=\left[H_{C}|h_{T_{i}}\right]$;28:     $n_{c}=n_{c}+1$;29:   **else**30:     $H_{C}=H_{C};$31:   **end if**32:**end for**33:Construct the parity-check matrix of the source code $H_{S}=\left[H_{T_{i}}\right]$ similarly according to lines 2 to 32.34:**End**


## 4. Simulation Results

**Figure 2:**
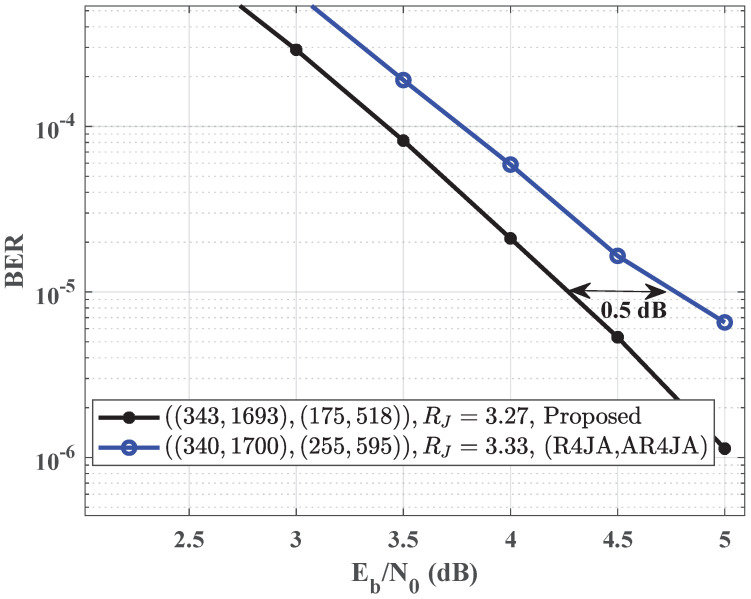
Comparison of BER performance between the proposed code pair and the code pair (R4JA, AR4JA) for p=0.01.

**Figure 3:**
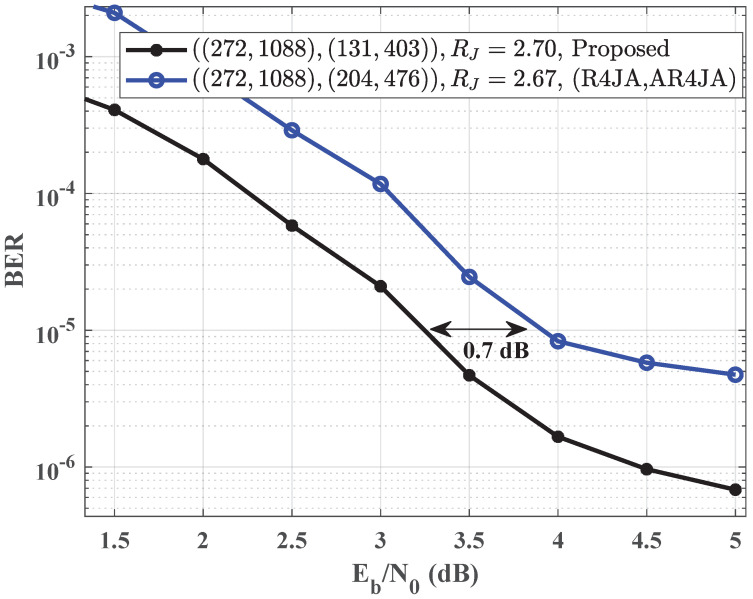
Comparison of BER performance between the proposed code pair and the code pair (R4JA, AR4JA) for p=0.015.

We evaluate the performance of the proposed JSCC system over AWGN channels in terms of bit error rate (BER) in this section. The simulation results are obtained by considering binary phase-shift keying modulation and employing joint belief propagation iterative decoding. The benchmark code pairs are generated using the progressive edge-growth algorithm with ‘copy-and-permute’ [23]. In contrast to the benchmark code pair, the channel code of the proposed code pair does not include punctured VNs. The complexity of proposed code pairs is primarily determined via the column weight of the proposed code, resulting in a complexity of $O(n^{W})$, where *W* is the larger value between the source code column weight $W_{s}$ and the channel code column weight $W_{c}$. The maximum number of iterations is set as 50, and that of erroneously decoded frames is set as 100 for all the signal-to-noise ratios (SNRs), denoted as $E_{b}/N_{0}$ in Figure 2 and Figure 3.

Figure 2 presents the simulated BER values for the benchmark code pair (repeat-by-4-jagged-accumulate (R4JA) [24], Accumulate-repeat-by-4-jagged-accumulate (AR4JA) [25]), and the proposed code pair according to Algorithm 1. The source and channel codes of the benchmark code pair are rate-1/5 R4JA and rate-2/3 AR4JA. The girth of the proposed code pair is 8, i.e., s=3. The column weights of the source and channel codes of the proposed code pair are $W_{s}=7$ and $W_{c}=8$, respectively. The frame length of the proposed code pair is 1693, and the corresponding frame length of the benchmark code pair (R4JA, AR4JA) is 1700. The code rates of the source and channel codes of the proposed code pair are 0.2 and 0.66. The code rate of the proposed code is slightly lower than that of (R4JA, AR4JA). Nevertheless, for p=0.01, the proposed code pair brings a 0.5 dB improvement over the benchmark code pair (R4JA, AR4JA) when the BER is at the level of $10^{-5}$.

Figure 3 shows the simulated BER performance of the code pair (R4JA, AR4JA) and the proposed code pair. The source and channel codes of the benchmark code pair are rate-1/4 R4JA and rate-2/3 AR4JA. The proposed code pair successfully avoids both length-4 and length-6 cycles. The column weights of the source and channel codes are $W_{s}=7$ and $W_{c}=7$. The code rates of the source and channel codes are 0.25 and 0.67. The girth of the proposed code pair is 8. In this study, the frame length of the proposed code pair is 1088, and the corresponding frame length of the benchmark code pair is 1088. Nevertheless, for p=0.015, the proposed code pair brings 0.7 dB gain over the benchmark code pair when the BER is at the level of $10^{-5}$.

**Figure 4:**
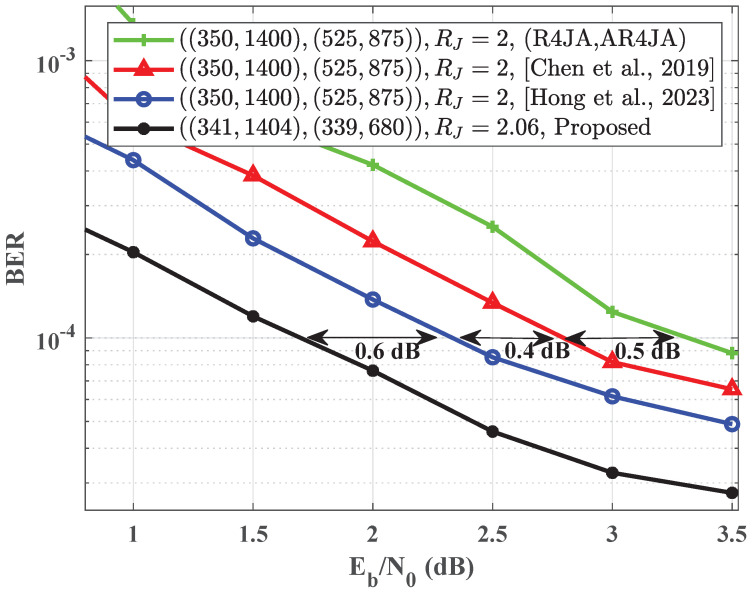
Comparison of BER performance involves the proposed code pair, the code pairs from [15,16], and the code pair (R4JA, AR4JA) for p=0.025.

Figure 4 presents the simulated BER performance of the proposed code pair alongside [15,16]. The code rates of the source and channel codes in [15,16] are 0.25 and 0.5, respectively. The proposed code pair effectively eliminates cycle of length-4 and length-6. The column weights of the source and channel codes are $W_{s}=8$ and $W_{c}=12$, respectively. The code rates of the source and channel codes are 0.24 and 0.5, respectively. The frame length of the proposed code pair is 1404, whereas the code pairs in [15,16] correspond to a frame length of 1400. When the probability *p* is set to 0.025, the proposed code pair outperforms the code pair in [15] by yielding a gain of 1 dB at the bit error rate of $10^{-4}$. Similarly, compared with the code pair in [16], the proposed code pair achieves a gain of 0.6 dB at the same bit error rate.

## 5. Conclusions

In this article, an algebraic construction method for LDPC code pairs of JSCC systems has been proposed based on the graph-theoretic approach. The constructed code pairs could avoid short-length cycles by following set constraints. The simulation results has shown that the proposed code pairs achieve significant performance improvement compared with the benchmark code pair (R4JA, AR4JA), [15,16]. The performance enhancement has been observed in both the waterfall region and the error-floor region. In addition, the proposed code pairs customized for the JSCC system have flexible frame lengths (i.e., enhanced adaptability over various channel conditions) unlike the protograph code pairs.

## Data Availability

The data that support the findings of this study are available from the corresponding author upon reasonable request.
